# Transcriptome and Allele Specificity Associated with a 3BL Locus for Fusarium Crown Rot Resistance in Bread Wheat

**DOI:** 10.1371/journal.pone.0113309

**Published:** 2014-11-18

**Authors:** Jian Ma, Jiri Stiller, Qiang Zhao, Qi Feng, Colin Cavanagh, Penghao Wang, Donald Gardiner, Frédéric Choulet, Catherine Feuillet, You-Liang Zheng, Yuming Wei, Guijun Yan, Bin Han, John M. Manners, Chunji Liu

**Affiliations:** 1 CSIRO Agriculture Flagship, St Lucia, QLD, Australia; 2 Triticeae Research Institute, Sichuan Agricultural University, Wenjiang, Chengdu, China; 3 National Center for Gene Research, Shanghai Institutes for Biological Sciences, Chinese Academy of Sciences, Shanghai, China; 4 CSIRO Agriculture Flagship, Black Mountain, ACT, Australia; 5 INRA-UBP Joint Research Unit 1095, Genetics, Diversity and Ecophysiology of Cereals, Clermont-Ferrand, France; 6 School of Plant Biology, Faculty of Science and The UWA Institute of Agriculture, The University of Western Australia, Perth, WA, Australia; University of Tasmania, Australia

## Abstract

Fusarium pathogens cause two major diseases in cereals, Fusarium crown rot (FCR) and head blight (FHB). A large-effect locus conferring resistance to FCR disease was previously located to chromosome arm 3BL (designated as *Qcrs-3B*) and several independent sets of near isogenic lines (NILs) have been developed for this locus. In this study, five sets of the NILs were used to examine transcriptional changes associated with the *Qcrs-3B* locus and to identify genes linked to the resistance locus as a step towards the isolation of the causative gene(s). Of the differentially expressed genes (DEGs) detected between the NILs, 12.7% was located on the single chromosome 3B. Of the expressed genes containing SNP (SNP-EGs) detected, 23.5% was mapped to this chromosome. Several of the DEGs and SNP-EGs are known to be involved in host-pathogen interactions, and a large number of the DEGs were among those detected for FHB in previous studies. Of the DEGs detected, 22 were mapped in the *Qcrs-3B* interval and they included eight which were detected in the resistant isolines only. The enrichment of DEG, and not necessarily those containing SNPs between the resistant and susceptible isolines, around the *Qcrs-3B* locus is suggestive of local regulation of this region by the resistance allele. Functions for 13 of these DEGs are known. Of the SNP-EGs, 28 were mapped in the *Qcrs-3B* interval and biological functions for 16 of them are known. These results provide insights into responses regulated by the 3BL locus and identify a tractable number of target genes for fine mapping and functional testing to identify the causative gene(s) at this QTL.

## Introduction

Fusarium pathogens cause two serious diseases in cereals, Fusarium crown rot (FCR) and Fusarium head blight (FHB). FCR is a chronic problem in many parts of the semi-arid cereal producing regions worldwide including Australia [1]. In contrast, FHB favours environments with high humidity and temperature. It is a sporadic problem in Australia but causes massive annual losses worldwide [2]. Both diseases can produce mycotoxins which can be harmful if present in foods or feeds [3], [4].

FHB is one of the most intensively studied diseases. Sources of resistance have been intensively searched [5], [6] and numerous quantitative trait loci (QTL) conferring resistance have been reported [3]. The best known source of FHB resistance is from the genotype Sumai 3 and the QTL on chromosome arm 3BS from this genotype is the most potent locus conferring resistance to this disease [7]. The 3BS locus contains a glycosyltransferase gene that has the potential to detoxify the mycotoxin deoxynivalenol which is also a virulence factor and this may explain the resistance mechanism [7]. This 3BS QTL does not confer any significant level of resistance to FCR in wheat [8]. In addition to the effort of map-based cloning of the 3BS resistance locus [9], transcriptome analysis has also been conducted in recent years to identify genes differentially expressed between FHB resistant and susceptible genotypes [10]–[15]. Efforts have also been made in transforming defence-related genes into susceptible or moderately susceptible wheat varieties to obtain transgenic plants with improved FHB resistance [16]–[19].

Compared with the studies on FHB, our knowledge of FCR and its possible resistance mechanisms is limited. There have been some studies on host transcriptional responses during the infection of susceptible genotypes following application of defence inducing compounds that can reduce FCR symptom development [20], [21]. Several QTL have been reported [22]–[26]. Among these, the one located on chromosome arm 3BL from the *Triticum spelta* accession ‘CSCR6’ (designated as *Qcrs-3B)* seems to be highly effective. This QTL accounted for up to 49% of phenotypic variance and provided significant effects in multiple hexaploid genetic backgrounds [24]. Recently, several independent sets of resistant and susceptible near-isogenic lines (NILs) that differ in the 3BL locus for FCR have been developed [27]. These genetic resources provide an ideal tool for studying the host responses to infection associated with resistance to this disease and for identifying genes that co-locate with the *Qcrs-3B* locus.

RNA sequencing (RNA-seq) has become a powerful tool for transcriptome analysis. The approach is not only highly sensitive and efficient for identifying differentially expressed genes (DEGs) [28] but, when combined with genomic and genetic analysis can also be used for detecting SNPs in transcribed genes that co-locate with a target locus [29]. These features of RNA-seq analysis are particularly attractive for applications in hexaploid wheat where multiple homoeologous alleles exist for most genes and transcripts. We thus conducted an RNA-seq analysis against five sets of the NILs developed for the *Qcrs-3B* locus [27], examined DEGs and SNPs between the NIL lines. This analysis provides candidate genes for both the response determined by the 3BL locus as well as those co-located with the 3BL locus that may be genetically causative for resistance. In addition to identifying genes underlying the FCR resistance locus, we were also interested in finding out if expressed genes associated with resistance to FCR were related to those observed by others for FHB.

## Materials and Methods

### Plant materials

Five sets of NILs generated using the heterogeneous inbred family method for the FCR QTL on chromosome arm 3BL reported by Ma et al. [27] were used in this study. Four of these NIL sets, including ‘1R/1S’, ‘2R/2S’, ‘3R/3S’, and ‘4R/4S’, were derived from the population of ‘Janz’*2/‘CSCR6’. The other set, ‘9R/9S’, was derived from the population of ‘Lang’/CSCR6’. ‘R’ isolines are those carrying the resistant allele and ‘S’ isolines are those carrying the susceptible allele at the *Qcrs-3B* locus. The FCR donor ‘CSCR6’ is a genotype belonging to the taxon *T. spelta* [24]. The NIL set ‘1R/1S’ (designated as Family A) was used for the primary analysis. The other four NIL sets (designated as Family B) were used for validating results obtained from Family A.

### Determination of the QTL *Qcrs-3B* chromosomal interval

**Figure 1:**
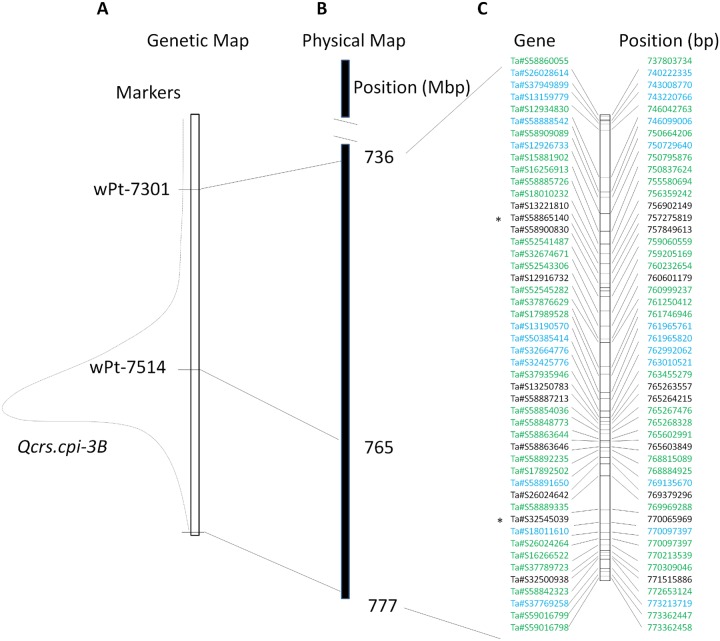
The *Qcrs-3B* interval and 48 unique genes identified in the interval. (A) Genetic map showing the *Qcrs-3B* interval with two DArT markers which were successfully placed on the 3B pseudomolecule of Chinese Spring. (B) The *Qcrs-3B* interval on the physical map of the 3B pseudomolecule; and (C) Genes located in the*Qcrs-3B* interval. Differentially expressed genes (DEGs) between the resistant and susceptible isolines were indicated as black for the up- and blue for the down-regulated genes. The expressed genes containing SNP were indicated as green. ‘*’ indicates that they were also DEGs.

The wheat 3B pseudomolecule ‘traes3bPseudomoleculeV1’of Chinese Spring was downloaded from Generic Genome Browser version 2.3 (https://urgi.versailles.inra.fr/gb2/gbrowse/wheat_annot_3B/) hosted by Unité de Recherche Génomique Info (URGI) in February, 2014 [30]. The DArT marker wPt-7301, which locates proximally to the QTL, was placed at about 736 Mb in the 3B pseudomolecule. Another marker, wPt-7514, which locates near the centre of the QTL was placed at about 765 Mb on the 3B pseudomolecule (Fig. 1). Sequences for the two DArT markers located distally to the QTL, however, were not available. Considering the terminal location of the QTL on this chromosome [24], the most distally located gene on the genomic sequence for this chromosome arm was used as the distal border of this QTL. Thus the size of the QTL interval used in this study could be over-estimated.

### Fusarium crown rot inoculation and experimental design

A single isolate of *F. pseudograminearum* (*Fp*, CS3427) was used in this study. This is one of the most aggressive isolates collected from northern New South Wales, Australia and maintained in the CSIRO collection [31]. Inoculum was prepared based on the method described before [32].

Surface-sterilized seeds were germinated in Petri dishes on three layers of filter paper saturated with water. Ten seedlings were used in each of the biological replications for all of the experiments conducted. Two-day-old seedlings were inoculated following the method described before [33] with the *F. pseudograminearum* isolate (*Fp*-infection) or water (mock). Samples were taken by cutting the shoot base (0–4 cm) at 3 or 5 days post inoculation (dpi) and frozen in liquid nitrogen immediately and kept at −80°C until processed.

Two datasets of RNA sequences were obtained in this study. The first dataset was obtained from Family A. The experimental design for Family A contained two treatments (mock and *Fp*-infection), two time points (3 and 5 dpi) and six biological replicates (Table S1). The second one was generated from Family B, which were used only to validate those expressed genes with SNPs (SNP-EGs) detected from Family A. A single trial with three biological replications was conducted for these four sets of NILs. Mocks were not used and samples were collected at 5 dpi only. Before RNA isolation, samples from the three biological replications for each of the eight isolines were pooled. Thus, a total of 8 samples (each for a different line of the four sets of NILs) were used for RNA extraction.

### RNA extraction, library construction and Illumina sequencing

Total RNA was isolated using a QIAGEN RNeasy plant mini kit (Qiagen, Hilden, Germany) according to the manufacturer’s instructions, using RLT buffer and including the optional on-column DNase I digestion. The yield and purity of each RNA sample was determined by the absorbance (Abs) at 260 and 280 nm and the integrity of all RNA samples was assessed on 1% agarose gels. Each sample of 10 µg of total RNA was sent to Australian Genome Research Facility Ltd (Parkville, Victoria, Australia) for further processing before Illumina HiSeq sequencing. Two technical replications were run for each of the 56 (48 for ‘1R/1S’ and 8 for the other 4 sets of NILs) cDNA libraries with 6 lanes of 100 bp paired-end sequencing. Raw reads were trimmed using a SolexaQA package 2.2 with minimum Phred quality value of 30 and minimum length of 70 bp. The RNA sequences were available at the National Center for Biotechnology Information (NCBI) with the accession number of SRP048912.

### Data quality control

For Family A, RNA-seq Illumina fastq sequence datasets were pooled by replicate (6 replicates per time point) at time points 3 dpi and 5 dpi. For Family B RNA sequences were pooled by response to FCR inoculation only (+/−) due to the fact that there was no biological replication used. FastQC (version 0.10.1) was used as a preliminary check that the Phred scores were acceptable. BioKanga (version 2.76.2; developed by bioinformatics team, unpublished) filtering was then employed and reads containing polymorphic variation but not supported by at least two other overlapping reads were removed from the datasets. Additionally, all but one instance of any duplicated reads were removed to reduce the effect of PCR artefacts. All retained reads for alignment were unique.

### RNA-seq analyses

Two methods were used in this study to analyse the trimmed RNA reads. One was based on the complete IWGSC (International Wheat Genome Sequencing Consortium) chromosome shotgun sequence contigs (CSS-contigs) (www.wheatgenome.org) due to their increased genome coverage for estimating distributions of DEGs and SNPs between the ‘R’ and ‘S’ isolines in the wheat genome. The other was based on UniGene (NCBI, www.ncbi.nlm.nih.gov/) aiming at identifying specific gene(s) underlying FCR resistance.

#### Distributions of DEGs and SNPs between chromosomes and on 3B

Following BioKanga (version 2.76.2) filtering, the retained reads were independently aligned against the complete CSS-contigs and the 3B pseudomolecule using BioKanga. Alignment parameters were set such that a maximum of two substitutions were allowed and no multi-aligned reads were accepted (except for reads mapping to the CSS once and the pseudomolecule). Two additional mismatches were allowed for the first 12 bp of the reads to account for primer artefact. Reads were aligned utilising the paired-end reads with insert sizes from 100 to 2 kbp. PCR differential amplification artefacts were reduced within the sequence alignment processing using BioKanga's sliding window mechanism. A counts matrix was generated and loaded into R (version 3.0.1) for downstream statistical analysis.

BioKanga SNP calling was run with raw, non-filtered reads aligned against the CSS contigs and 3B pseudomolecule allowing at most two mismatches and at most two additional mismatches for the first 12 bp of the reads accounting for primer artefact. Only unique alignments were accepted. A custom R function was written to identify candidate SNPs underlying the QTL. This method applied multiple criteria to identify trait linked SNPs which identified polymorphisms between the two isolines for a given set of NILs across replicates and was able to detect presence or absence polymorphism. The criteria used for identification of SNPs included: (1) The loci present in all replicates must be homozygous (the dominating nucleotide must have a ratio higher than 0.9; (2) any candidate SNP must have had a minimum of 3 reads coverage. Each candidate SNP was visually checked using the Integrative Genomics Viewer (version 2.3.12).

#### UniGene-based analysis of DEGs and SNPs

Analysis of gene expression was performed using BioKanga. Reference sequences used in this analysis included 58,596 of the wheat UniGenes [downloaded from ftp://ftp.ncbi.nih.gov/repository/UniGene/Triticum_aestivum/of NCBI in Jun of 2012] and the genome sequences of the diploid wheat A-genome progenitor *T. urartu* and the diploid D-genome progenitor *Aegilops tauschii* (downloaded from NCBI) [34], [35].

The obtained reads were aligned against the 58 K UniGenes and the diploid A and B genome reference sequences with no more than two mismatches allowed. Only those reads matching best with the wheat UniGenes were selected for expression analysis. BioKanga ‘maploci’ was used to normalize counts based on RPKM (Reads per kilobase per million reads). Prior to the differential expression analysis, the 12 replicates (6 biological and 2 technical) for each genotype-treatment-timepoint sample were merged together for a given pair of comparison by BioKanga genDEseq. In total, four pairwise comparisons between genotypes were conducted. These are summarised throughout the paper in the following way: S^M^3_v_R^M^3; S^M^5_v_R^M^5; S^I^3_v_R^I^3 and S^I^5_v_R^I^5. Symbols are ‘M’ for mock; ‘I’ for *Fp-infection*; ‘3’ for 3 dpi; ‘5’ for 5 dpi; ‘R’ for the resistant isoline R; ‘S’ for the susceptible isoline S, and ‘a_v_b’ for comparing object ‘a’ with ‘b’, in which ‘a’ is the control and ‘b’ is the treatment. DEGs were determined with the threshold of FDR≤0.01 and the absolute value of log_2_FoldChange ≥1 or ≤−1 or ‘inf’ (the value of one comparative object is zero and the other is not). The R software (version 3.1.0) was used to generate heat maps.

Genes responsive to FCR infection were identified by four pairwise comparisons between the treatments: R^M^3_v_R^I^3; R^M^5_v_R^I^5; S^M^3_v_S^I^3; S^M^5_v_S^I^3. The responsive genes after *Fp*-infection compared with mock were identified with the same method as DEGs: threshold of FDR≤0.01 and the absolute value of log_2_FoldChange ≥1 or ≤−1 or ‘inf’.

The trimmed sequences for each line-treatment-timepoint sample were pooled together. SNPs between 4 pairwise comparisons were identified: S^M^3_v_R^M^3, S^M^5_v_R^M^5, S^I^3_v_R^I^3, and S^I^5_v_R^I^5. The alignment of reads to the reference sequences was performed with a maximum of 2 mismatches per read. Minimum coverage for the declaration of an SNP was 4 nucleotides. SNPs between the resistant and susceptible isolines were identified using the Biokanga snpmarkers sub-process with a minimum 90% score, i.e., the percentage of a given nucleotide at a SNP position is at least 90% in the resistant or susceptible isolines.

DEGs and expressed genes containing SNP (SNP-EGs) was mapped in the targeted QTL interval by blasting them against the 3B pseudomolecue. All sequence comparisons were performed using the BLASTN 2.2.26+ algorithm with e-value <10^−5^ and length >100 bp.

### Functional annotation

Functions of UniGenes were annotated using the Blast2GO program (version 2.6.6) with default parameters except that e-value threshold of 10^−10^ was used when executing steps of ‘Blast’ and ‘Annotation’. Alignments with a higher score were visually inspected and annotated if a reasonable degree of homology was observed.

### Validation of DEGs by real-time qualitative PCR

Among the identified DEGs between the ‘R’ and ‘S’ isolines from Family A, a total of 4 genes were randomly selected and assessed using real-time quantitative PCR (RT-qPCR) given that six biological replicates were used for RNA-seq analysis. *Ta.27922.1.S1_x_at*, encoding a cyclin family protein, was used as the internal reference gene [36]. Primers were designed based on the tool of Primer-BLAST (http://www.ncbi.nlm.nih.gov/tools/primer-blast/) and listed in Table S2. The validations were conducted using Family A. FCR inoculation, tissue sampling and RNA extraction were based on the methods as described earlier and three biological replications were used. RNA extraction, cDNA synthesis, and expression analyses were carried out as described by Ma et al. [37]. Each biological replication was analysed in two separate wells (technical replication). The average values from the two replications were used for each biological replication. Calculations of the relative fold change were conducted using the method of 2−ΔΔ*C*T. Transcripts with Ct values >40 cycles were regarded as having no expression value.

### Validation of SNP-EGs by re-sequencing

Three genes with SNPs, *Ta#S37789723*, *Ta#S52545282*, and *Ta#S58887817*, were randomly selected for validation by re-sequencing. Primers were designed based on alignments between wheat UniGene and sequences of *T. urartu* and *Ae. tauschii* (Table S2). Genomic DNA from the isolines ‘1R’ and ‘1S’ were extracted from 20-day old seedlings using the hexadecyltrimethylammonium bromide (CTAB) method [38]. PCR amplification and sequencing were conducted based on the methods described by Ma et al. [39] with annealing temperatures ranging from 62°C to 65°C depending on the primers (Table S2).

## Results

### Exploratory analysis of variance factors in gene expression patterns

A total of 152 Gb sequences were obtained from Family A and 77 Gb sequences from Family B. Prior to fitting models, some basic exploratory analysis was conducted looking at the variance components for both Family A and B based on sequence reads. All samples were consistent and no outliers were observed. For Family A, the largest proportion of the variance was driven by time point. Principal component analysis (PCA) demonstrated that the samples may be separated by the first two components according to the time point and the genotype (Figs. S1A and S1B). The hierarchical clustering (Fig. S1C) was consistent with the PCA. For Family B, the experimental run effect was quite strong. However, a large proportion of the variance still contributed to the genotypic effect (data not shown).

### Distribution of DEGs and SNPs in the wheat genome

**Figure 2:**
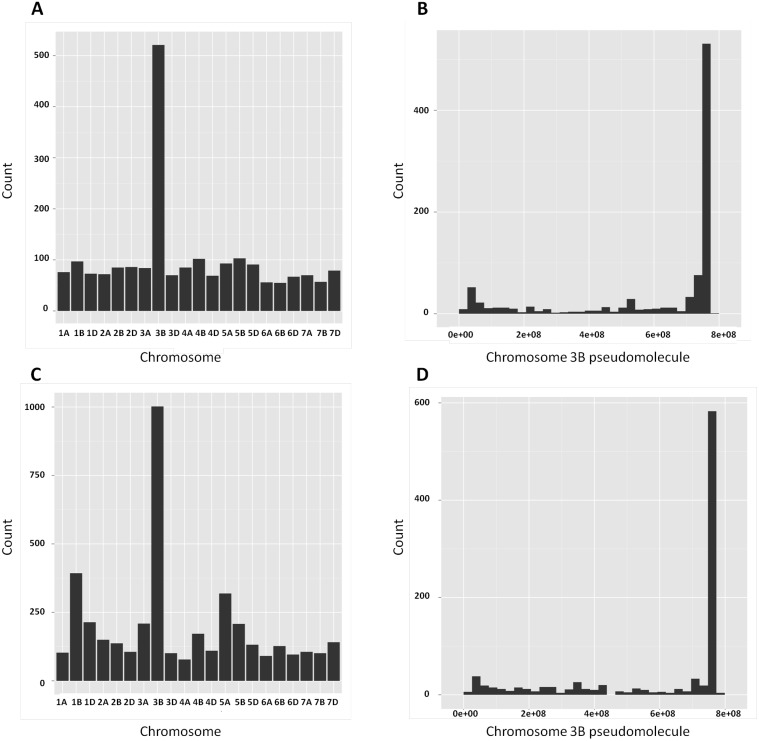
Distribution of DEGs (A, B) and SNPs (C, D) across the 21 chromosomes (left) and along the 3B pseudomolecule (right). Note: 3B pseudomolecule is from short (left) to long (right) arm in base pair (bp).

For increased genome coverage, the exploratory analysis on distribution of the detected DEGs and SNP-EGs in the wheat genome was conducted using the CSS contigs. This analysis detected 2,020 DEGs between the two isolines of Family A. Chromosome 3B had significantly more DEGs compared with other chromosomes families (Fig. 2A) and that a large proportion of those mapped on chromosome 3B was concentrated on the distal end where the targeted QTL resides (Fig. 2B).

Based on the use of the CSS contigs, 955 SNPs were detected between the ‘R’ and ‘S’ isolines of Family A and chromosome 3B had significantly more SNPs compared with other chromosomes (Fig. 2C) and that a large proportion of those on chromosome 3B co-located with the targeted QTL at the distal end of this chromosome arm (Fig. 2D).

### Genes induced by *Fusarium* infection

**Figure 3:**
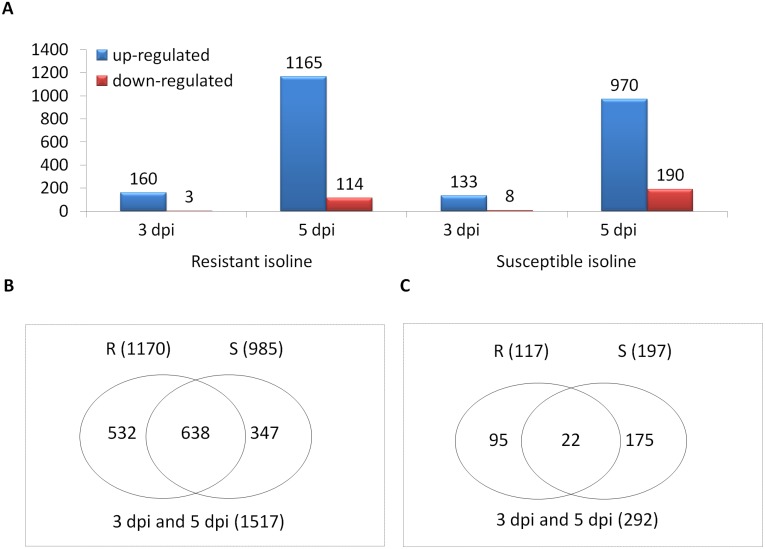
Differentially expressed genes (DEGs) between the resistant and susceptible isolines in Family A following *Fusarium pseudograminearum* (*Fp) –*infection (compared to those in the mock). (A) Overview of the number of DEGs. (B) Venn diagrams showing the number of up- or (C) down-regulated genes in the resistant isoline compared with those in the susceptible isoline. DEGs were determined with the threshold of FDR ≤0.01 and the absolute value of log_2_FoldChange ≥1 or ≤−1 or ‘inf’ (the value of one comparative object is zero and the other one is not). Symbols are ‘M’ for mock; ‘I’ for *Fp*-infection; ‘R’ for resistant isoline; and ‘S’ for susceptible isoline.

Only data from Family A were suitable for this analysis as ‘mock’ controls were not used in assessing the NILs of Family B. Following *Fp*-inoculation, the numbers of up-regulated genes detected from the ‘R’ lines were 160 at 3 dpi and 1,165 at 5 dpi; and from the ‘S’ lines were 133 and 970, respectively, at the two different time points. The numbers of down-regulated genes detected at the two different time points following *Fp*-inoculation were 3 and 114, respectively, from the ‘R’ line, and 8 and 190, respectively, from the ‘S’ line (Fig. 3A).

**Figure 4:**
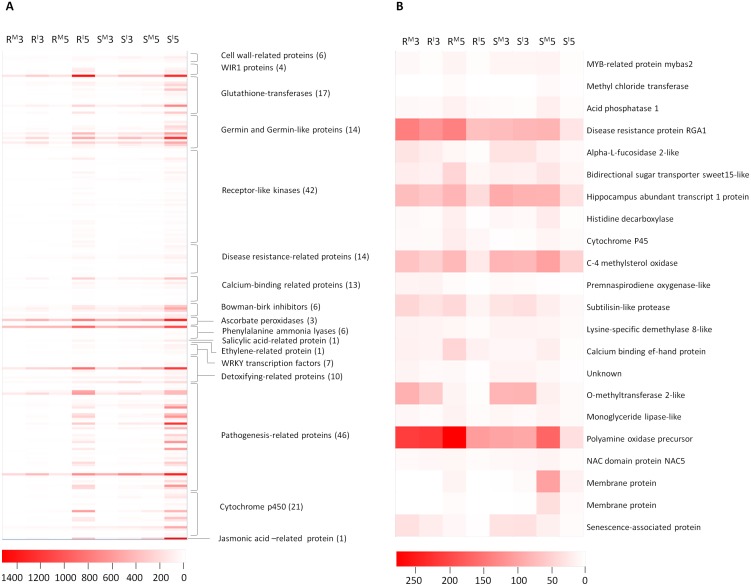
Heat maps showing up- (A) or down - regulated genes (B) belonging to various classes in Family A detected between *pseudograminearum* infection (*Fp-*infection) and water treatment (mocks) at 3 dpi and 5 dpi, respectively. Up- or down-regulated genes were determined with the threshold of FDR≤0.01 and the absolute value of log_2_ FoldChange ≥1 or ≤−1 or ‘inf’ (the value of one comparative object is zero and the other one is not). Symbols are ‘M’ for mock; ‘I’ for *Fp-*infection; ‘3’ for 3 dpi; ‘5’ for 5 dpi; ‘R’ for resistant isoline; ‘S’ for susceptible isoline. The color key represents the RPKM normalized value. Lighter color indicates greater transcript accumulation. Each column represents a sample and each row a gene.

In total, 1,809 induced genes (1,517 up- and 292 down-regulated) were detected between the two isolines following *Fp*-infection (Fig. 3B and 3C). Of them, 638 were up-regulated and 22 down-regulated in both isolines (Table S3). The 638 up-regulated genes contain 46 encoding pathogenesis-related proteins, 42 encoding receptor-like kinases, 21 encoding cytochrome P450 s, 17 encoding glutathione transferases, and 10 encoding detoxifying-related proteins (Fig. 4A). They also contain genes encoding proteins involved in host-pathogen interactions: 14 for disease resistance-related proteins, 6 for cell wall-related proteins, 4 for WIR1 (wheat induced resistance 1) proteins, 7 for WRKY transcription factors, 3 for ascorbate peroxidises, 6 for phenylalanine ammonia lyases, and 14 for germin and germin-like proteins (Fig. 4A). They also contain genes for the biosynthesis of plant hormones which are known to be involved in wheat-*Fusarium* interactions: jasmonic acid (JA), ethylene (ET) and salicylic acid (SA) (Fig. 4A). Three of the 22 down-regulated genes seem to be highly relevant to disease resistance as they encode disease resistance protein RGA1, an EF-hand calcium binding protein, and a senescence-associated protein (Fig. 4B). Fifty-two of the up-regulated genes and three of the down-regulated ones co-located with the targeted QTL interval (Table S3), and five of them were among those showing differences between the ‘R’ and ‘S’ lines (below).

### Transcriptome differences between resistant and susceptible isolines

**Figure 5:**
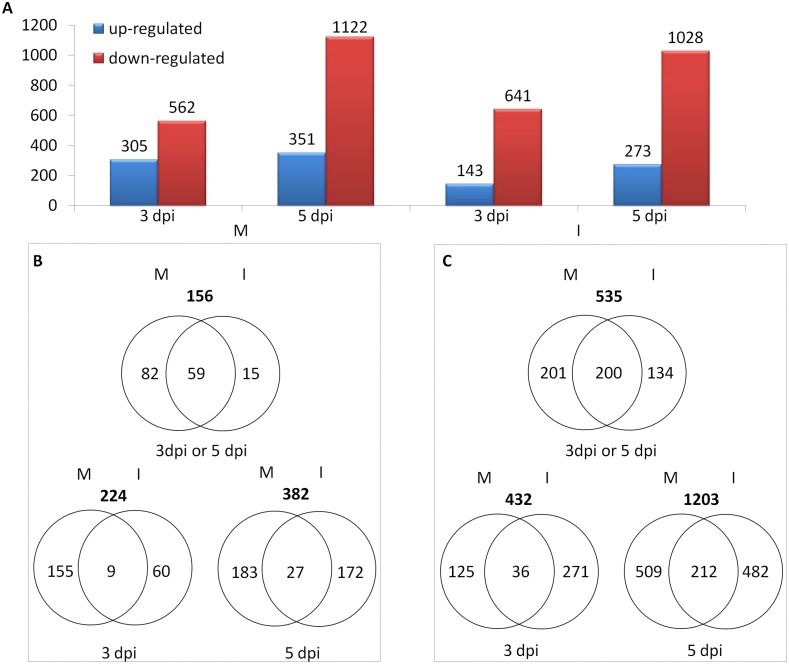
Genes responsive to *Fusarium pseudograminearum* (*Fp*) infection. (A) Numbers of genes exhibiting differential accumulation between *Fp*-infected and mock plants at either 3 dpi or 5 dpi. (B) Venn diagrams showing the number of up-regulated or (C) down-regulated genes. These genes were determined with the threshold of FDR≤0.01 and the absolute value of log2FoldChange ≥1 or ≤−1 or ‘inf’ (the value of one comparative object is zero and the other one is not). Symbols are ‘M’ for mock; ‘I’ for *Fp*-infection; ‘R’ for resistant isoline; ‘S’ for susceptible isoline.

An important part of this investigation was to identify transcripts that are differentially expressed between the ‘R’ isoline and the ‘S ‘isoline to provide an indication of what molecular mechanisms may be associated with resistance. The numbers of DEGs from the ‘mock’ treatment in Family A were: 562 down-regulated and 305 up-regulated at 3 dpi and 1,122 down-regulated and 351 up-regulated at 5 dpi. The numbers of DEGs detected from the *Fp* treatment between the NILs were: 641 down-regulated and 143 up-regulated at 3 dpi; and 1,028 down-regulated and 273 up-regulated at 5 dpi (Fig. 5). The DEGs obtained between the two treatments at the two time points assessed between the two isolines represent a total of 691 (156 up- and 535 down-regulated in the ‘R’ isoline) unique transcripts (Fig. 5 and Table S4).

Of these 691 genes differentially expressed between the ‘R’ and ‘S’ lines, 88 (12.7%) were derived from chromosome 3B. Twenty-two (10 up- and 12 down-regulated) of them on chromosome 3B were mapped in the *Qcrs-3B* interval (Table S5). Functions for 6 of the 10 up-regulated genes and 7 of the down-regulated genes are known (Table S5).

The 691 DEGs also contained 8 genes which were detected from the resistant isolines only and they were absent in the ‘S’ isoline of Family A assessed (Table 1). Biological functions are known for only one of these 8 genes. That is *Ta#S32500938*, encoding the gibberellin 2-beta-dioxygenase 8-like. Two of the 8 DEGs (*Ta#S12916732* and *Ta#S32500938*) were mapped in the *Qcrs-3B* interval with *Ta#S32500938* being the only one annotated with known biological function.

**Table 1:** Genes expressed in the resistant isoline only^a^.

Unigene name	Annotation^b^	Expression value^c^							
		S^M^3	R^M^3	S^M^5	R^M^5	S^I^3	R^I^3	S^I^5	R^I^5
Ta#S12916732	Unknown	0	3.3	0	3.3	0	3.1	0	2.9
Ta#S58856178	Unknown	0	11.8	0	14.6	0	9.8	0	9.7
Ta#S58856166	Unknown	0	14	0	14.7	0	11.8	0	14.5
Ta#S58850454	Unknown	0	5.5	0	5.5	0	4.8	0	4.4
Ta#S32500938	Gibberellin2-beta-dioxygenase 8-like	0	2.4	0	1.4	0	3.3	0	2.7
Ta#S16236095	Unknown	0	2.1	0	3.5	0	3.2	0	2.8
Ta#S58857100	Unknown	0	4.6	0	6.1	0	3.5	0	2.6
Ta#S58856177	Unknown	0	3.7	0	3.2	0	3	0	4.7

a Genes were identified with the threshold of FDR≤0.01.

b Annotations were performed using the software Blast2Go with BLASTX E-score of less than 10^−10^.

c RPKM normalized values are shown. The symbols are: ‘M’ for mock; ‘I’ for *Fp*-infection; ‘3’ for 3 dpi; ‘5’ for 5 dpi; ‘R’ for resistant isoline; ‘S’ for susceptible isoline.

### Expressed genes containing SNPs (SNP-EGs) between the NILs

**Figure 6:**
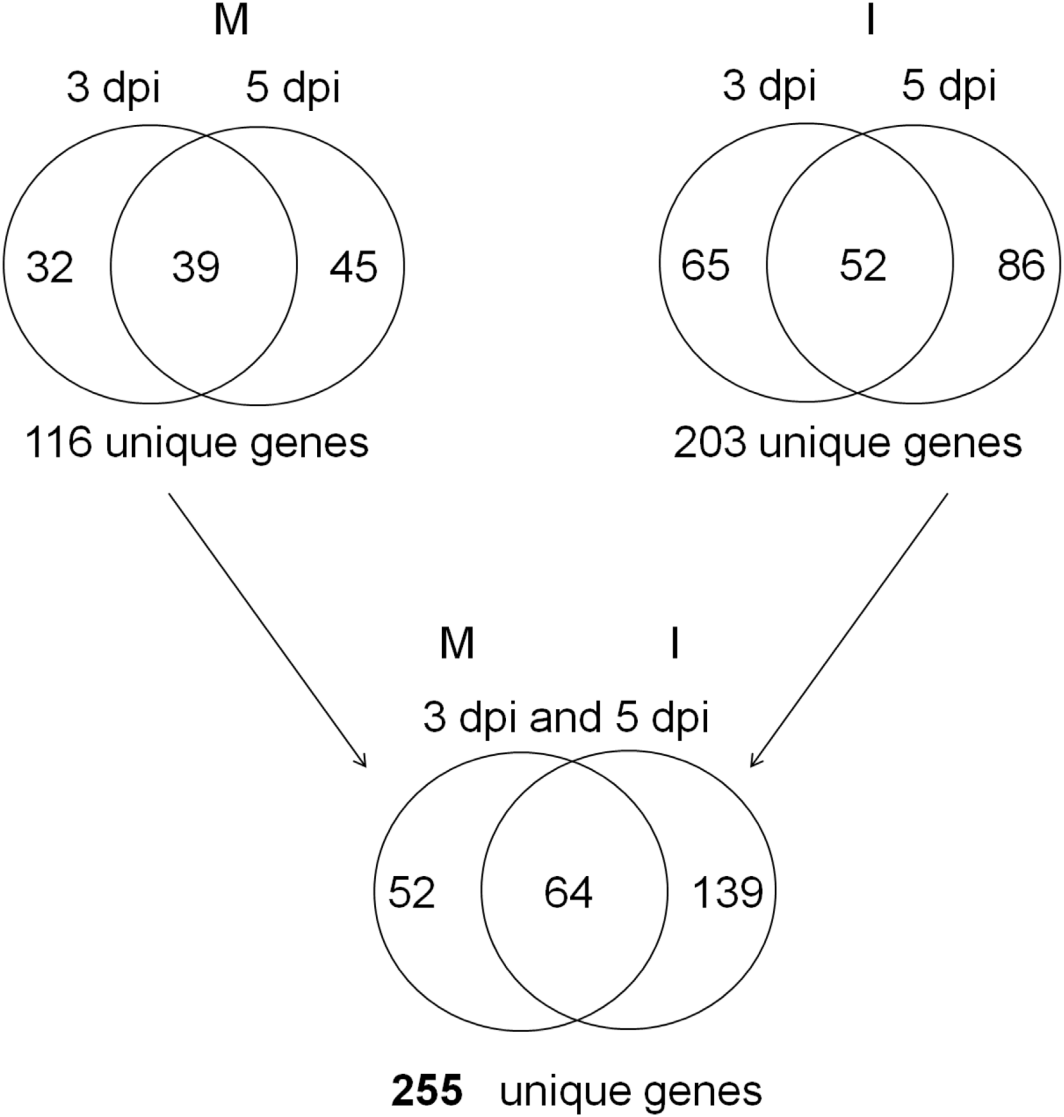
Venn diagrams showing the number of the expressed genes with SNPs between the resistant and susceptible isolines of Family A (1R/1 S). Symbols are ‘M’ for mock or water-treatment; ‘I’ for *Fp*-infection; ‘3’ for 3 dpi and ‘5’ for 5 dpi.

A total of 255 unique SNP-EGs between the NIL set ‘1R/1S’ of Family A were detected. Among them, 203 were detected from the *Fp*-infected samples, 116 from the mock samples, and 64 were detected from both of the samples (Fig. 6). Of these 255 unique SNP-EGs, 60 (or 23.5%) were mapped on chromosome 3B, and 28 genes with a total of 71 SNPs were mapped in the *Qcrs-3B* interval (Table S6). Biological functions for 16 of these 28 genes are known (Table S6).

Of the 28 SNP-EGs mapped in the targeted QTL interval in the Family A, 18 (64.3%) were also detected among the four sets of NILs in Family B. These 18 SNP-EGs contain a total of 56 SNPs. Forty-one of these 56 SNPs (73.2%) were among those detected in Family A (Tables 2 and S6).

**Table 2:** Validation of SNP-EGs and SNPs identified from Family A in Family B.

NILs	SNP-EGs	SNPs	Novel SNPs
2R/2S	14	32	9
3R/3S	13	28	7
4R/4S	11	25	8
9R/9S	14	29	7
Unique	18	41	15

### Validation of DEGs and SNP-EGs detected from the RNA sequence analysis

To verify the RNA-seq results obtained, RT-qPCR analysis was conducted against 4 genes that were randomly selected from the DEGs between the ‘R’ and ‘S’ isolines detected based on UniGene analysis. The expression patterns of these 4 genes assessed by RT-qPCR (Fig. S2) were consistent with those obtained from the RNA-seq analysis.

Three of the genes with a total of 10 SNPs identified between the resistant and susceptible isolines were also randomly selected for validation based on re-sequencing the NIL set ‘1R/1S’. All of the SNPs were identified correctly in the re-sequencing experiment (Fig. S3).

## Discussion

RNA-seq analysis was conducted against five sets of NILs for a large-effect locus conferring FCR resistance on chromosome arm 3BL in wheat. Not unexpectedly, the numbers of detected SNP-EGs from these NILs on the targeted chromosome 3B were significantly higher compared with those on any other chromosome and a large proportion of them were concentrated on the distal end of chromosome arm 3BL where the targeted *Qcrs-3B* locus locates. Interestingly, this was also observed for DEGs between ‘R’ and ‘S’ isolines suggesting that there may be a regulation of proximally located genes by the *Qcrs-3B* locus. The use of the multiple sets of NILs allowed the identification of better defined sets of candidate genes underlying the targeted locus. Functions of these genes provide insights into responses regulated by the 3BL locus and the 48 genes mapped in the targeted QTL interval are tractable in further efforts to functionally test gene(s) underlying this QTL for causal effects. These genes are being used as markers in fine mapping the *Qcrs-3B* locus based on a NIL-derived population.

### Candidate genes underlying the *Qcrs-3B* QTL for FCR resistance

Of the large numbers of DEGs and SNP-EGs detected in this study, targeting those located in the *Qcrs-3B* interval could be productive in further efforts of characterizing the FCR locus and cloning genes underlying the QTL. The 22 DEGs and the 28 SNP-EGs represent a total of 48 unique genes as two of the DEGs also contain SNPs (Fig. 1).

Five of the 22 DEGs mapped in the targeted QTL interval do not only differ between the ‘R’ and ‘S’ lines but were also induced by *Fp*-infection (Table S6). Several of these DEGs have been previously associated with host-pathogen interactions. They include those encoding the pathogenesis-related protein 4, the disease resistance response protein 206-like, and the disease resistance protein RPM1. One of the up-regulated genes mapped in the *Qcrs-3B* interval encodes a homologue of resistance protein RGA2, which is known to confer broad spectrum resistance to potato late blight [40].

The 22 DEGs mapped in the targeted QTL interval also include two of those expressed in the resistant isolines only. Of these two genes, only *Ta#S32500938* was annotated with known biological function. This gene encodes a gibberellin 2-beta-dioxygenase 8-like enzyme. It degrades active gibberellin and is involved in cold response [41]. Clearly, together with the other one gene expressed in the resistant isolines only and located in the targeted QTL interval (*Ta#S12916732*), possible roles of *Ta#S32500938* in FCR resistance need to be clarified.

Of the 28 SNP-EGs located in the targeted interval, only two were significantly differentially expressed between the ‘R’ and ‘S’ isolines assessed. Considering that a difference in a gene sequence does not necessarily result in significantly changed expression but could affect the expression of down-stream genes [37], the gene(s) underlying the *Qcrs-3B* could also be among the 18 non-DEGs located in the QTL interval. Out of these SNP-EGs, three encode MYB transcription factors which have been previously implicated in host-pathogen interactions [42]. Another one of the 28 SNP-EGs with known functions is particularly interesting as it encodes an NADH-quinone oxidoreductase subunit which is a well-known defence protein involved in detoxification reaction [43].

### DEGs induced by FCR and FHB infections

There is a plethora of transcriptomic studies on FHB in recent years [15], [44], [45] and it is known that all *Fusarium* pathogens which cause FHB can cause FCR [1]. Considering the shared aetiology, pathogen biology and epidemiology between FHB and FCR [1], a comparison of host genes induced between these two diseases could be interesting.

As a transcriptomic analysis routinely detects hundreds of DEGs, it is not unexpected that such a comparison identified many genes which were induced by both FHB and FCR. Genes responsive to both diseases include those encoding some well-known defence proteins [e.g. pathogenesis-related (PR) proteins, receptor-like kinases (RLKs), glutathione-S-transferases, and cytochrome P450s] [20], [21], [32], [46], [47], those encoding responsive proteins during disease infection, those encoding xylanase inhibitors that can hinder the xylanase released by *Fusarium* pathogens from degrading the primary component of cell walls [48], and those of WRKY transcription factors which are known to function in biotic and abiotic stress responses [49]. The shared genes induced by these two diseases also include those encoding detoxifying-related proteins involved in detoxifying and transporting mycotoxins produced by *Fusarium* species [50], [51], and those encoding germ and germin-like proteins that are related to metabolism of reactive oxygen species (ROS) [52], [53]. Such ROS as superoxide and hydrogen peroxide can induce programmed cell death (PCD) [54]. Another gene induced by both FCR and FHB was one of those encoding PCD-related proteins [12]. Similarly, two of the DEGs detected between the ‘R’ and ‘S’ lines of FCR in this study, *Ta#*S22386683 and *Ta#S37823230,* were among those detected between the NILs for a FHB locus [12].

When focused on the targeted QTL interval, however, overlap of genes detected between FHB and FCR was not obvious. For example, none of the DEGs located near the *Fhb1* locus using the deletion line [15] was among those located in the *Qcrs-3B* region in this study. This is not difficult to comprehend in considering that different DEGs were not only detected between NILs for different loci of FHB [44], [45] but also detected for the same *Fhb1* locus between the use of NILs [44] and a deletion line [15].

## Supporting Information

Figure S1 **Principal component analysis (PCA) of RNA sequences from Family A by time point (A), genotype (B) and hierarchical clustering with Genotype×Treatment×Timepoint (C).** IDs for each database were listed in Table S1.
(TIF)

Figure S2 **RT-qPCR validation of 4 genes showing differential expression between the resistant and susceptible isolines of the NIL set ‘1R/1S’.** The columns represented the average expression ratios calculated from all three biological replications. For *TA#S58850454*, no expression was detected in the susceptible isoline. The values were presented as the averages of 2∧ (Ct_TA#S58850454_-Ct_reference gene_). Error bar shows standard deviation. Symbols are ‘M’ for mock; ‘I’ for *Fp*-infection, ‘3’ for 3 dpi and ‘5’ for 5 dpi.
(TIF)

Figure S3 **Multiple alignments of** ***Ta#S37789723*** **(A),** ***Ta#S52545282*** **(B) and** ***Ta#S58887817*** **(C) with sequences from the resistant and susceptible isolines.** The SNPs in the red box were validated. Those in black box were identified by re-sequencing.
(TIF)

Table S1 **Sample summary of the first set of the near isogenic lines (Family A).**
(XLSX)

Table S2 **Primers used for real-time qualitative PCR analysis and for the validation of single nucleotide polymorphism detected from the RNA sequence analysis.**
(XLSX)

Table S3 **Transcripts that exhibited differential accumulation in both the resistant and the susceptible isolines following** ***Fusarium pseudograminearum*** **(** ***Fp*** **) treatment compared to those in the mock at 3 dpi and 5 dpi.**
(XLSX)

Table S4 **Differential expressed genes (DEGs) in the resistant isoline compared with the susceptible isoline of Family A following** ***Fusarium pseudograminearum*** **(** ***Fp*** **) or water (mock) treatment at 3 dpi or 5 dpi.**
(XLSX)

Table S5 **Differentially expressed genes (DEGs) between the resistant and susceptible isolines that mapped in the** ***Qcrs-3B*** **interval.**
(XLSX)

Table S6 **Genes with homozygous SNPs between the resistant and susceptible isolines mapped in the** ***Qcrs-3B*** **interval.**
(XLSX)
