# Supplementary figures and images for: Transcriptome and Allele Specificity Associated with a 3BL Locus for Fusarium Crown Rot Resistance in Bread Wheat
Source: PLoS One. 2014 Nov 18;9(11):e113309. doi: 10.1371/journal.pone.0113309 (PMC4236173; doi:10.1371/journal.pone.0113309)

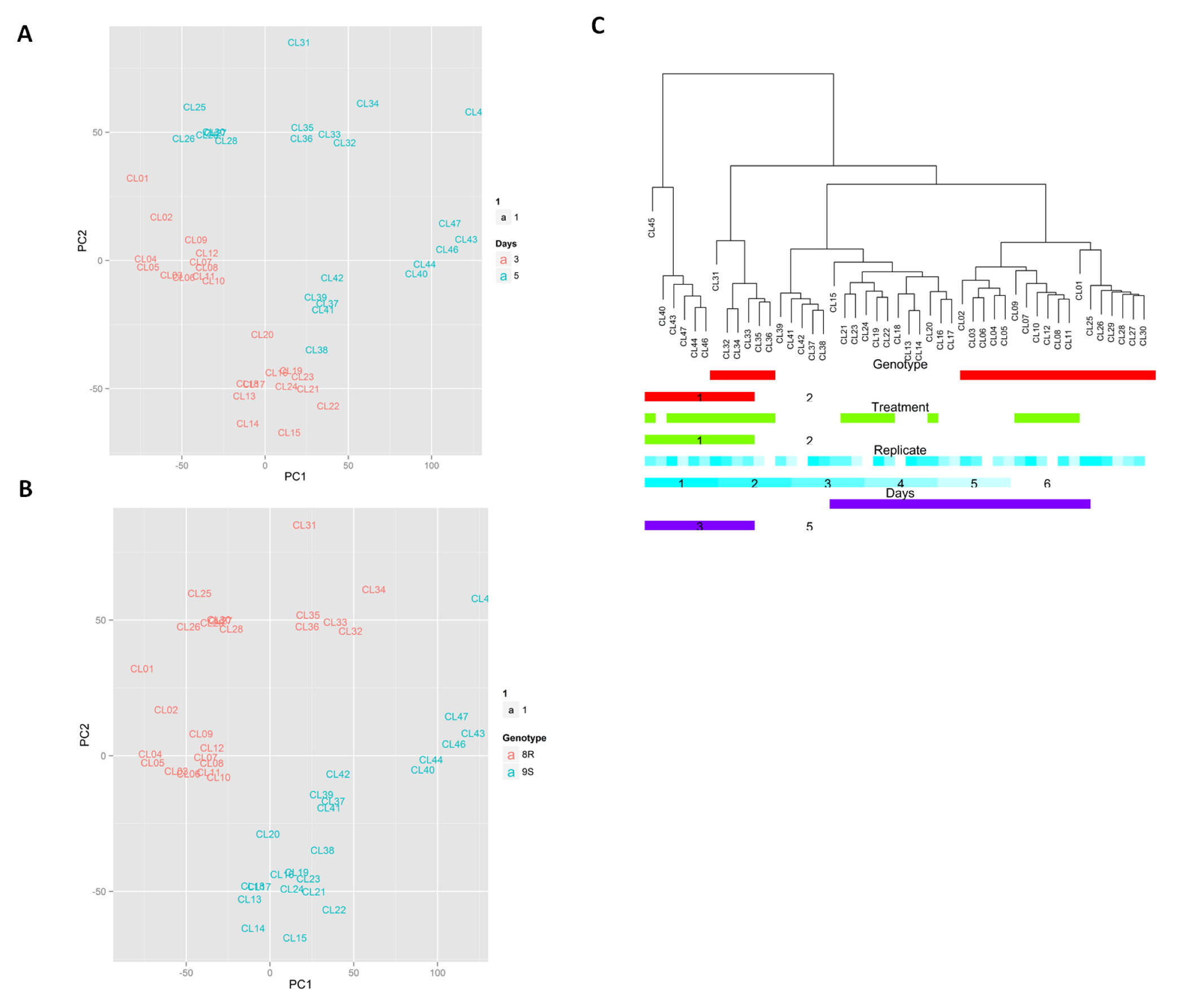

Supplement: Figure S1 — Principal component analysis (PCA) of RNA sequences from Family A by time point (A), genotype (B) and hierarchical clustering with Genotype×Treatment×Timepoint (C). IDs for each database were listed in Table S1. (TIF) [file pone.0113309.s001.tif]

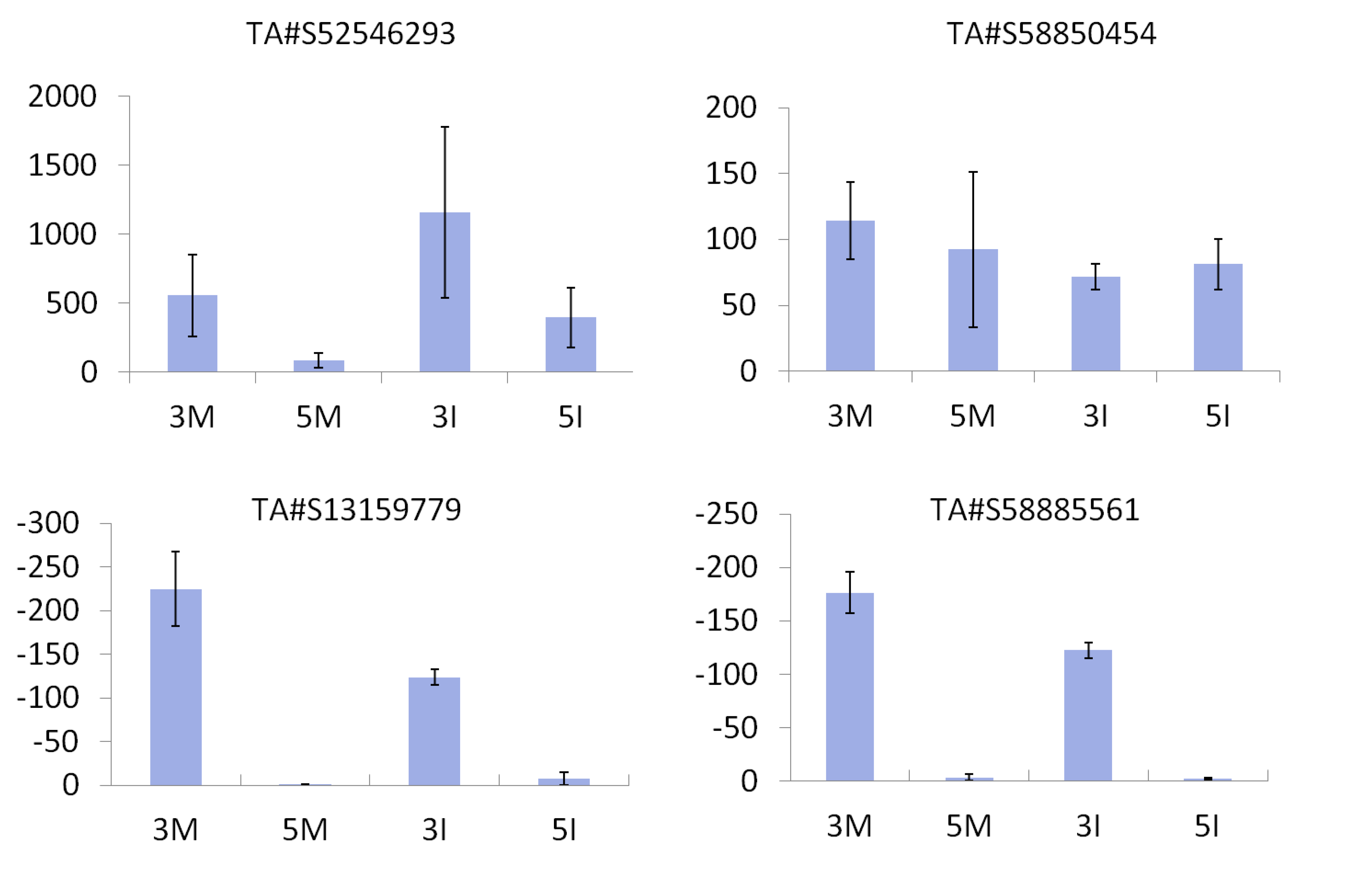

Supplement: Figure S2 — RT-qPCR validation of 4 genes showing differential expression between the resistant and susceptible isolines of the NIL set ‘1R/1S’. The columns represented the average expression ratios calculated from all three biological replications. For TA#S58850454, no expression was detected in the susceptible isoline. The values were presented as the averages of 2∧ (CtTA#S58850454-Ctreference gene). Error bar shows standard deviation. Symbols are ‘M’ for mock; ‘I’ for Fp-infection, ‘3’ for 3 dpi and ‘5’ for 5 dpi. (TIF) [file pone.0113309.s002.tif]

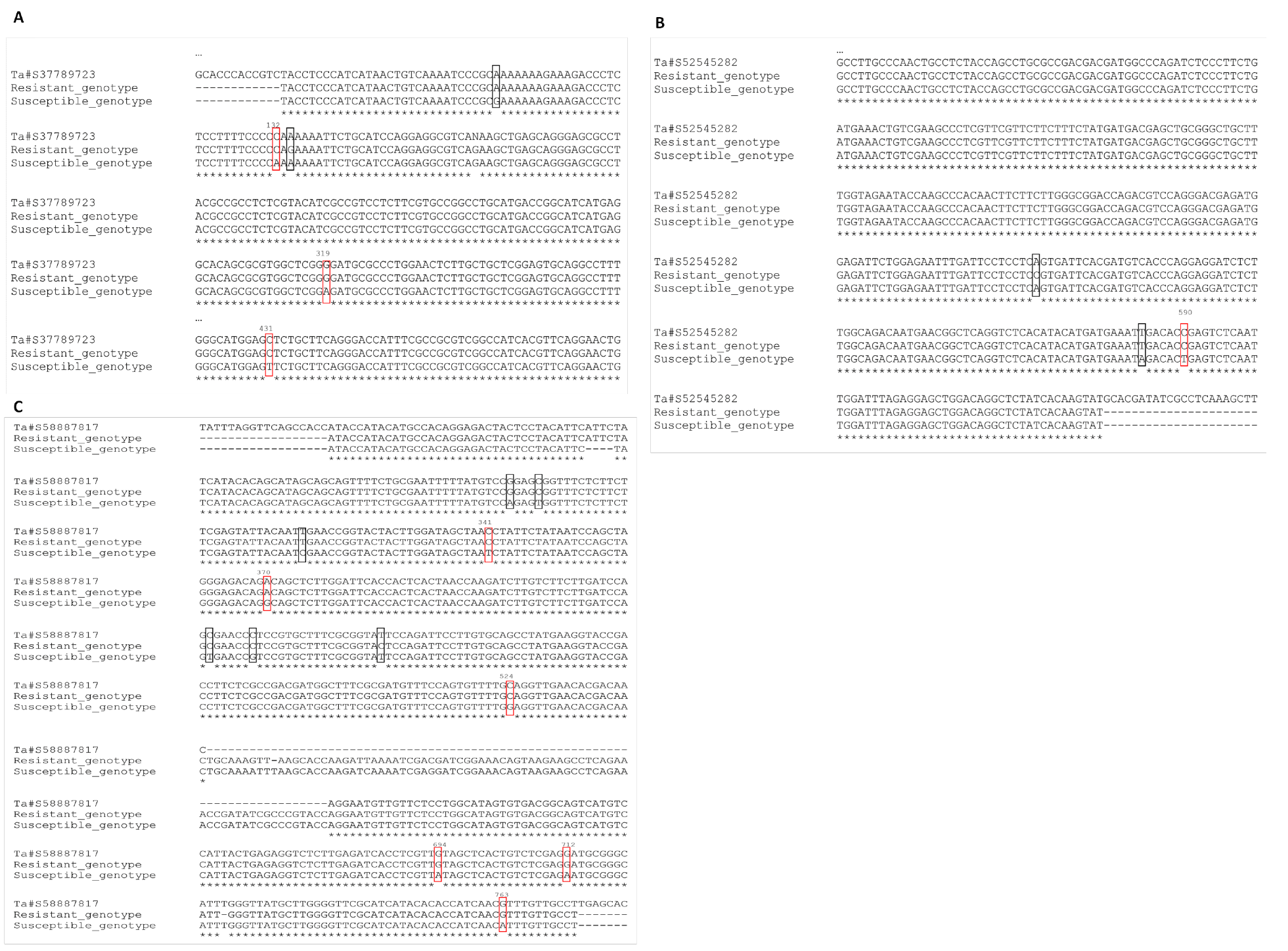

Supplement: Figure S3 — Multiple alignments of Ta#S37789723 (A), Ta#S52545282 (B) and Ta#S58887817 (C) with sequences from the resistant and susceptible isolines. The SNPs in the red box were validated. Those in black box were identified by re-sequencing. (TIF) [file pone.0113309.s003.tif]
